# Effects of C-terminal amidation and heptapeptide ring on the biological activities and advanced structure of amurin-9KY, a novel antimicrobial peptide identified from the brown frog, *Rana kunyuensis*

**DOI:** 10.24272/j.issn.2095-8137.2018.070

**Published:** 2019-05-18

**Authors:** Fen Zhang, Zhi-Lai Guo, Yan Chen, Li Li, Hai-Ning Yu, Yi-Peng Wang

**Affiliations:** 1 Department of Pharmaceutical Sciences, College of Pharmaceutical Sciences, Soochow University, Suzhou Jiangsu 215123, China; 2 Department of Bioscience and Biotechnology, Dalian University of Technology, Dalian Liaoning 116023, China; 3 School of Life Sciences, Guizhou Normal University, Guiyang Guizhou 550001, China

**Keywords:** Antimicrobial peptides, *Rana kunyuensis*, Amurin-9KY, Heptapeptide ring, C-terminal amidation, Structure activity relationship

## Abstract

*Rana kunyuensis* is a species of brown frog that lives exclusively on Kunyu Mountain, Yantai, China. In the current study, a 279-bp cDNA sequence encoding a novel antimicrobial peptide (AMP), designated as amurin-9KY, was cloned from synthesized double-strand skin cDNA of *R. kunyuensis*. The amurin-9KY precursor was composed of 62 amino acid (aa) residues, whereas the mature peptide was composed of 14 aa and contained two cysteines forming a C-terminal heptapeptide ring (Rana box domain) and an amidated C-terminus. These structural characters represent a novel amphibian AMP family. Although amurin-9KY exhibited high similarity to the already identified amurin-9AM from *R. amurensis*, little is known about the structures and activities of amurin-9 family AMPs so far. Therefore, amurin-9KY and its three derivatives (amurin-9KY1–3) were designed and synthesized. The structures and activities were examined to evaluate the influence of C-terminal amidation and the heptapeptide ring on the activities and structure of amurin-9KY. Results indicated that C-terminal amidation was essential for antimicrobial activity, whereas both C-terminal amidation and the heptapeptide ring played roles in the low hemolytic activity. Circular dichroism (CD) spectra showed that the four peptides adopted an α-helical conformation in THF/H_2_O (v/v 1:1) solution, but a random coil in aqueous solution. Elimination of the C-terminal heptapeptide ring generated two free cysteine residues with unpaired thiol groups, which greatly increased the concentration-dependent anti-oxidant activity. Scanning electron microscopy (SEM) was also performed to determine the possible bactericidal mechanisms.

## INTRODUCTION

Antimicrobial peptides (AMPs), which are small, cationic and amphipathic peptides widely distributed throughout organisms, are evolutionarily ancient weapons against environmental pathogens (Zasloff, 2002). They are important components of innate immune systems and play key roles in the anti-infective immune responses of organisms (Radek & Gallo, 2007). According to previous studies, AMPs possess strong and diverse antimicrobial activities against bacteria, fungi, viruses and even protozoa. The activities and specificities of AMPs can be affected by various factors such as molecular weight, sequence, charge, conformation, hydrophobicity and amphipathicity (Brogden, 2005). The antimicrobial mechanisms are diverse among different AMPs. However, most AMPs are thought to function by forming pores in the membranes of target microorganisms, ultimately leading to disruption of cellular integrity (Nicolas, 2009).

Frogs belonging to the family Ranidae are widely distributed around the world, except for the Polar Regions, southern South America and most of Australia (Conlon et al., 2004). So far, 347 ranid species have been identified worldwide, with most living in moist environments surrounded by diverse pathogens. As a result, they are continuously threatened by pathogenic invasions and have therefore evolved effective immune systems (Conlon et al., 2004; Zasloff, 2002). Among these systems, AMPs play key roles in the anti-infective immune response of ranid frogs (Kreil, 1994). To date, hundreds of AMPs have been characterized from various ranid species. According to their primary structures, they are divided into dozens of different families, including gaegurins, brevinins (1 and 2), ranalexins, ranatuerins (1 and 2), esculentins (1 and 2), palustrins, japonicins (1 and 2), nigrocin-2, tigerinins, temporins and odorranains (A–W) (Conlon, 2004; Duda et al., 2002; He et al., 2012; Li et al., 2007; Matutte et al., 2000).

Brown frogs, also known as wood frogs, are a group of ranid species belonging to *Rana*. Currently, there are 14 recognized brown frogs in China distributed in 30 provinces. *Rana kunyuensis*, an endemic brown frog species in China, exclusively lives on Kunyu Mountain, Yantai, Shandong Province. In the present study, we identified a novel AMP family member, named amurin-9KY, from the skin secretions of *R. kunyuensis*. The structure-function relationship of amurin-9KY was clarified to establish the effect of C-terminal amidation and the heptapeptide ring on the biological activities and advanced structures of amphibian AMPs.

## MATERIALS AND METHODS

### Frog collection

Adult specimens of *R. kunyuensis* (*n*=5, weight range 5–10 g) were captured on Kunyu Mountain, Yantai, Shandong Province, China. The frogs were housed in plastic box and fed with yellow mealworms in the lab for one week until the experiment.

### Total RNA extraction, cDNA synthesis and amurin-9KY- encoding cDNA screening

An individual frog (female, 8 g) was washed with distilled water twice and anaesthetized with ice before being euthanized with a needle. The dorsal skin was quickly removed with a small pair of scissors and placed into a pre-cooling homogenizer. The skin was then immediately homogenized into powder in liquid nitrogen and mixed with Trizol reagent (Invitrogen, CA, USA). The subsequent procedures were carried out according to the manufacturer’s instructions and the extracted total RNA was preserved in liquid nitrogen until use. All animal experimental protocols in the present study were approved by the Animal Care and Use Ethics Committee of Soochow University.

Double-strand cDNA was synthesized using an In-Fusion SMARTer^TM^ Directional cDNA Library Construction Kit (Clontech, USA). The primers used for first strand synthesis were 3${}^{\prime }$ In-Fusion SMARTer CDS Primer, 5${}^{\prime }$-CGGGGTACG ATGAGACACCA(T)_20_VN-3${}^{\prime }$ (N=A, C, G, or T; V=A, G, or C) and SMARTer V Oligonucleotide, 5${}^{\prime }$-AAGCAGTGGTATCAA CGCAGAGTACXXXXX-3${}^{\prime }$ (X=undisclosed base in the proprietary SMARTer oligo sequence). The enzyme used for first strand synthesis was SMARTScribe^TM^ Reverse Transcriptase and was supplied by the kit. The second strand was amplified by 50× Advantage 2 Polymerase Mix using 5${}^{\prime }$ PCR Primer II A and 3${}^{\prime }$ In-Fusion SMARTer PCR Primer, 5${}^{\prime }$-CGGGGTACGATGAGACACCA-3${}^{\prime }$. The synthesized double-strand cDNA was stored at –80 ${}^{\circ }$C until use.

The synthesized double-strand cDNA was used as a template to screen the cDNAs encoding the AMPs of *R. kunyuensis*. A sense degenerate oligonucleotide primer (RanaAMP, 5${}^{\prime }$-CCAAAGATGTTSMCCWYGAAG-3${}^{\prime }$, M=A or C; W=A or T; Y=C or T), designed according to the conserved signal peptide domain sequences of previously characterized AMPs from the skin of ranid frogs, and a 3${}^{\prime }$-antisense primer (3${}^{\prime }$-PCR, 5${}^{\prime }$-CGGGGTACGATGAGACACCAT-3${}^{\prime }$) were used for PCR analysis. The PCR procedure was as follows: 4 min of denaturation at 95 ${}^{\circ }$C; 30 cycles: denaturation at 95 ${}^{\circ }$C for 30 s, annealing at 57 ${}^{\circ }$C for 30 s, extension at 72 ${}^{\circ }$C for 1 min; final extension at 72 ${}^{\circ }$C for 10 min. The PCR product was purified by agarose gel electrophoresis, cloned into pMD^TM^19-T vector (Takara, Dalian, China) and transformed into *E. coli* for sequencing.

### Peptide synthesis

Amurin-9KY and its derivatives (amurin-9KY1–3) were chemically synthesized on an Applied Biosystems model 433A peptide synthesizer (ABI, USA) according to the manufacturer’s standard protocols. The crude peptides were purified by reversed-phase high performance liquid chromatography (RP-HPLC) to a final purity higher than 95% and their identities were confirmed by automated Edman degradation and matrix-assisted laser desorption/ionization–time-of-flight mass spectrometry (MALDI-TOF-MS). The intra-peptide disulfide bridge and N-terminal amidation were accurately formed in the synthesis process.

### Antimicrobial assay

In total, eight microbial strains, including gram-positive bacteria, gram-negative bacteria and fungi, were used for the antimicrobial assay. The assays included inhibition zone examination and minimum inhibitory concentration (MIC) determination and were conducted as described in our previous papers (He et al., 2012; Lu et al., 2010). Briefly, microbes were inoculated in Mueller-Hinton broth (MH) and incubated at 37 ${}^{\circ }$C to the exponential phase. The inoculum was then diluted with fresh MH broth to 10^6^ CFU/mL, and 50-μL bacterial dilutions were mixed with serial dilutions of peptides (50 μL) in 96-well microtiter plates. The plates were incubated at 37 ${}^{\circ }$C for 18 h and the minimum concentrations at which no visible growth of bacteria occurred were recorded as MIC values.

### Hemolytic assay

Hemolytic assay was conducted as previously reported (Wang et al., 2011). Fresh human erythrocytes were collected from a healthy donor and washed with 0.9% saline. The erythrocytes were re-suspended with 0.9% saline and incubated with serial peptide dilutions at 37 ${}^{\circ }$C for 30 min. The mixtures were then centrifuged at 2 000 r/min at room temperature for 5 min, after which the supernatants were removed and their absorbance was measured at 540 nm. We used 1% (v/v) Triton X-100 to determine the 100% hemolysis, with 0.9% saline used as the negative control.

### Anti-oxidant assay

As a stable nitrogen radical, 2, 2-diphenyl-1-picrylhydrazyl (DPPH, Sigma, USA) has been widely used to examine the anti-oxidant activities of biological samples, drugs and foods. In the present study, the DPPH radical scavenging activities of the peptides were determined according to our previously described method (Zhang et al., 2012). Briefly, 92 μL of 6×10^−5^ mol/L DPPH radical dissolved in methanol was mixed with 8 μL of peptide solution (2 mg/mL, mass ratio of peptide to DPPH of ∼3:1). The mixture was kept in the dark for 30 min at room temperature and then the amount of reduced DPPH was quantified by measuring the decrease in absorbance at 520 nm. The DPPH radical scavenging percentage (S%) of the peptides was calculated according to the formula: S%=(A_blank_−A_sample_)×100/A_blank_. Deionized water was used as the negative control.

### Circular dichroism (CD) spectroscopy

To investigate the secondary structure of amurin-9KY and its derivatives (amurin-9KY1–3), CD spectroscopy was performed using a Jasco J-810 spectrophotometer (JASCO, Japan). Samples with a constant peptide concentration of 0.12 mg/mL were prepared in two different solvents, i.e., water and 50% (v/v) trifluoroethanol (TFE)-water, and added to a quartz optical cell with a path length of 0.1 cm at 25 ${}^{\circ }$C. The spectra were averaged over three consecutive scans, followed by subtraction of the CD signal of the solvent.

### Scanning electron microscopy (SEM)

The surface morphologies of the AMP-treated microbes were observed by SEM, which can partly reveal the antimicrobial mechanisms of AMPs. In the present study, *S. aureus* ATCC25923 was used to evaluate the potential antimicrobial mechanism of amurin-9KY. The experiment was carried out according to the method described by Lu et al. (2010). The concentration of amurin-9KY used in the experiment was 1×MIC. The treatment conditions of the bacterial and amurin-9KY mixture were 37 ${}^{\circ }$C for 30 min. After that, samples were prepared, and photographs were taken. Ampicillin was used as the positive control in the experiment.

## RESULTS

### cDNA cloning and characterization of amurin-9KY

**Figure 1:**
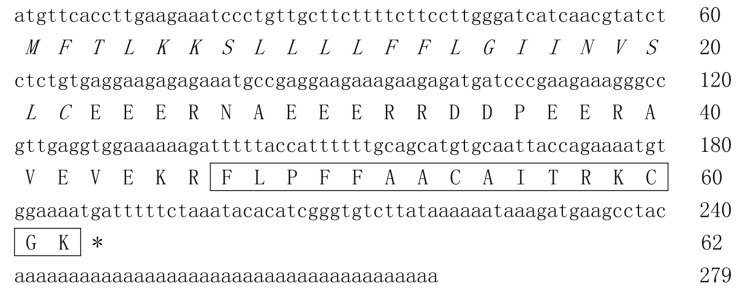
Nucleic acid sequence of cDNA encoding the precursor of amurin-9KY

A 279-bp cDNA sequence (GenBank accession No.: JX421759) encoding a 62-aa precursor was cloned from the constructed skin cDNA library of *R. kunyuensis*. The nucleotide sequence of the cDNA and the deduced amino acid sequence are shown in Figure 1. To confirm the existence and accuracy of the cloned cDNA sequence, two antisense specific primers, that is, RKAMP-R1 (5${}^{\prime }$-GCCAAGACACCCGATGTGTATTTAG-3${}^{\prime }$) and RKAMP-R2 (5${}^{\prime }$-CCCTTTTCCACATTTTCTGGTAATT-3${}^{\prime }$), were designed according to the cloned cDNA sequence and coupled with the sense specific primer RanaAMP for PCR (synthesized skin cDNA library of *R. kunyuensis* was used as a template). The PCR products were sequenced and verified the identity of the cDNA sequence above (data not shown).

The signal peptide domain is indicated in italics, the putative mature peptide is boxed and the stop codon is indicated by an asterisk (*).

The 62-aa AMP precursor deduced from the cDNA sequence included an N-terminal signal peptide domain of 22 aa, an acidic amino acid residue-rich (Asp and Glu) spacer peptide domain and a mature peptide domain of 16 aa residues. There was a conserved dibasic cleavage site Lys-Arg (K-R) between the spacer peptide domain and mature peptide domain, which was likely cleaved by trypsin-like proteases followed by mature peptide release. Sequence alignment of the mature peptide with the NCBI protein database revealed that it possessed highest primary structure similarity with amurin-9AM (GenBank accession No.: AEP84587). Hence, it was designated amurin-9KY according to the origin of *R. kunyuensis*.

**Figure 2:**
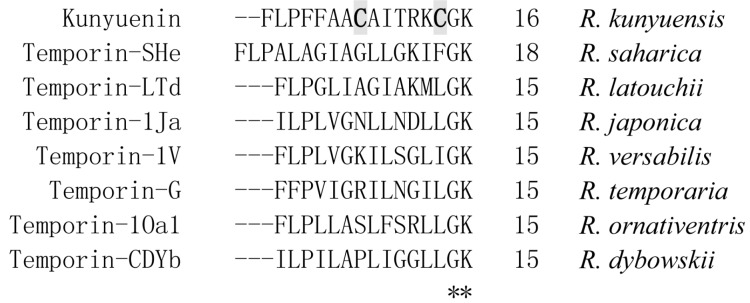
Amino acid sequence comparison of amurin-9KY with amurin-9AM from *R. amurensis* and temporin family AMPs from other ranid frogs

Amurin-9KY was also found to possess high sequence similarity to the temporin AMP family, especially the C-terminal Gly-Lys (G-K) di-residue, which is regarded as an amidation site (Figure 2). However, unlike the temporin family, amurin-9KY exhibited two cysteine residues forming an intra-molecular disulfide bridge, which appeared as a C-terminal heptapeptide ring (Rana box domain). The physical and chemical parameters of amurin-9KY were computed by ProtParam (http://web.expasy.org/protparam/), demonstrating a molecular weight of 1 584.97, theoretical pI of 8.96 and net charge of +2.

All AMP sequences used were derived from the NCBI protein database. The two cysteine residues of amurin-9KY are indicated in gray shadow. The length of the AMPs is shown and species from which the AMPs are derived are on the right. Asterisks (*) indicate identical residues.

To evaluate the influence of C-terminal amidation and the heptapeptide ring on the biological activities of the peptide *in vitro*, three derivatives (amurin-9KY1–3) were designed and chemically synthesized, with their biological functions then examined (Table 1).

**Table 1:** Structural parameters of amurin-9KY and its derivat

Sample	Amino acid sequence	Molecular weight	Structure characteristics
Amurin-9KY	FLPFFAACAITRKC-NH_2_	1 584.96	C-terminal amidation and C-terminal heptapeptide ring C8-C14
Amurin-9KY1	FLPFFAACAITRKC-NH_2_	1 586.96	C-terminal amidation
Amurin-9KY2	FLPFFAACAITRKC	1 585.96	C-terminal heptapeptide ring C8-C14
Amurin-9KY3	FLPFFAACAITRKC	1 587.96	None

### Antimicrobial and hemolytic assays

As listed in Table 2, amurin-9KY was active against the tested gram-positive bacteria *S. aureus* ATCC25923, S. *aureus* 090223^+^ (IS) and *N. asteroids* 090312^+^ (IS), and showed the strongest potency against *S. aureus* ATCC25923, with an MIC of 4.68 μg/mL. However, amurin-9KY did not show any antimicrobial activity against the tested gram-negative bacteria or fungi, consistent with the features of the temporin family (Conlon, 2004). The derivative amurin-9KY1 exhibited less potent activities against all three gram-positive bacteria. Interestingly, unlike amurin-9KY, amurin-9KY1 was able to kill the eukaryotic microbe *Slime mold* 090413 (IS) with an MIC value of 75 μg/mL. However, derivatives amurin-9KY2 and amurin-9KY3 exhibited no activities against any of the tested microbes.

**Table 2:** Antimicrobial activities of amurin-9KY and its derivatives

	MIC (μg/mL)			
**Microorganism**	Amurin-	Amurin-	Amurin-	Amurin-
	9KY	9KY1	9KY2	9KY3
**Gram-positive**				
*Staphylococcus aureus* ATCC25923	4.68	37.5	ND	ND
*Staphylococcus aureus* 090223^+^ (IS)	37.5	75	ND	ND
*Nocardia asteroids* 090312^+^ (IS)	37.5	75	ND	ND
**Gram-negative**				
*Escherichia coli* ATCC25922	ND	ND	ND	ND
*Klebsiella pneumonia* 1368 (IS)	ND	ND	ND	ND
*Pseudomonas aeruginosa* ATCC27853	ND	ND	ND	ND
**Fungi**				
*Candida albicans* ATCC2002	ND	ND	ND	ND
*Slime mold* 090413 (IS)	ND	75	ND	ND

MIC: Minimum inhibitory concentration. These concentrations represent mean values of three independent experiments performed in duplicate. IS: Clinically isolated strain. ND: No detectable activity in inhibition zone assay at a dose of 2 mg/mL.

The hemolytic activities of amurin-9KY and the three derivatives were also examined. All four peptides showed slight hemolytic activity against fresh human erythrocytes at concentrations of 100 μg/mL, with hemolytic rates of 2%, 15.4%, 17.9% and 20.8%, respectively.

### Anti-oxidant activity

**Figure 3:**
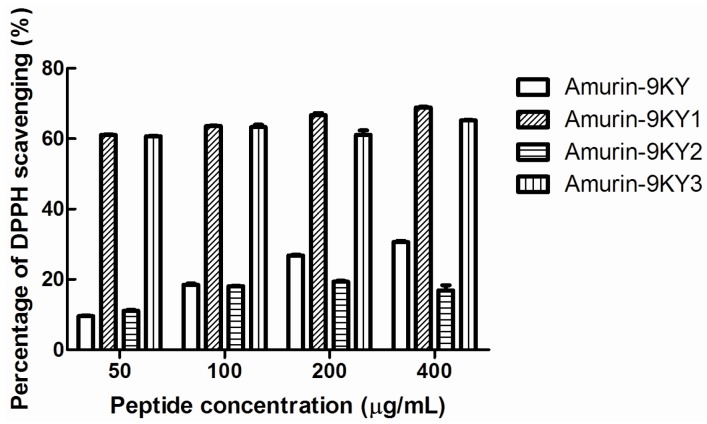
Anti-oxidant activity of amurin-9KY and derivatives(amurin-9KY1–3)

To date, many peptides exhibiting anti-oxidant activity have been identified from several species of ranid frogs, which constitute the excellent skin anti-radiation defense system of ranid frogs (He et al., 2012; Liu et al., 2010; Lu et al., 2010; Yang et al., 2009). Most are dual-functional peptides, possessing both anti-oxidant and antimicrobial activities (He et al., 2012; Liu et al., 2010; Lu et al., 2010; Yang et al., 2009). In the present study, the anti-oxidant activities of amurin-9KY and the three derivatives were also evaluated (Figure 3). Amurin-9KY exhibited slight concentration-dependent DPPH radical scavenging activity, with an optimal S% value of 30.6% at a concentration of 400 μg/mL. In contrast, the derivatives amurin-9KY1 and amurin-9KY3 exhibited strong anti-oxidant activities, with S% values exceeding 60% at concentrations as low as 50 μg/mL. Compared to the other three peptides, amurin-9KY2 exhibited the lowest anti-oxidant activity, with an S% value of 20% at a concentration of 400 μg/mL.

Different concentrations of peptides were incubated with DPPH solution at room temperature for 30 min, and then the amount of reduced DPPH was quantified by measuring the decrease in absorbance at 520 nm. Results represent mean values of three independent experiments.

### Solution structures of amurin-9KYs

**Figure 4:**
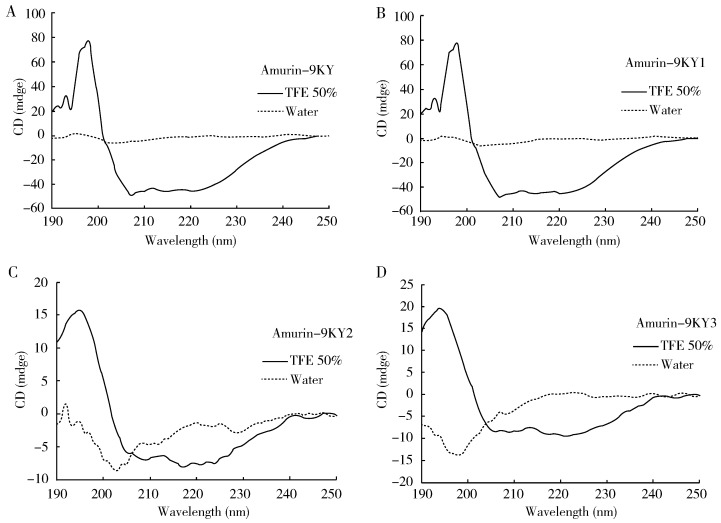
CD spectra of the four peptides in different solutions

The CD spectra of amurin-9KY in water showed a negative band at 200 nm, indicating a random-coil conformation. In the membrane-mimetic solvent (50% TFE-water) the presence of one positive band (190 nm) and two large negative dichroic bands at 208 and 222 nm (–50 mdeg) in amurin-9KY was consistent with the α-helical conformations (Figure 4A). For amurin-9KY1–3, the negative dichroic bands at 208 and 222 nm were much smaller, about –10 mdeg. The α-helical structure of most active AMPs is thought to be responsible for the formation of pores in the membranes of target organisms, thus disrupting metabolic activity (Brogden, 2005). The CD results support the concept that amurin-9KY most likely killed the bacteria through membrane disruption. In addition, the much smaller negative 208 and 222 nm dichroic bands were in good agreement with the antimicrobial activity data for amurin-9KY1–3.

A: Amurin-9KY; B–D: Amurin-9KY1–3.

### Effects of amurin-9KY on microbial membrane morphology by SEM

**Figure 5:**
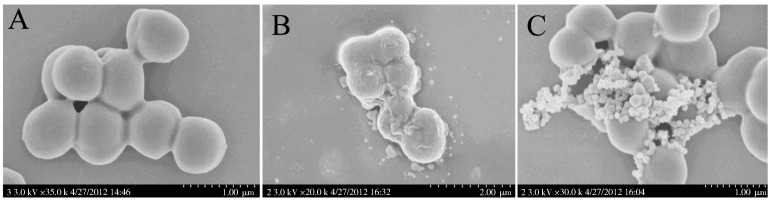
Scanning electron micrographs of *S. aureus* treated with amurin-9KY

Previous studies have demonstrated that AMPs achieve antimicrobial activity by disrupting various key microorganism cell processes, with some AMPs possessing multiple mechanisms (Brogden, 2005; Nguyen et al., 2011). There are many specific targets in microbial cells for AMPs, including external proteins, outer surface lipids, outer membrane proteins (gram-negative bacteria), inner membrane, integral membrane proteins, nucleic acids and intracellular proteins (Liu et al., 2017, 2018; Nguyen et al., 2011). Among them, disrupting the integrity of the microbial inner membrane is the most common mode for AMPs (Liu et al., 2017, 2018; Nguyen et al., 2011), the disruption of which results in obvious morphological alteration. Here, the antimicrobial results showed that *S. aureus* ATCC25923 was most sensitive to amurin-9KY, and therefore it was selected to examine the induced membrane morphological alterations by SEM. As illustrated in Figure 5, the untreated *S. aureus* cells exhibited normal shape and smooth surfaces (Figure 5A). In contrast, after treatment with amurin-9KY for 30 min, the cellular shape and surface of *S. aureus* exhibited obvious alterations (Figure 5B). The bacterial cells showed rough surfaces, prevalent membrane vesicles, and cellular fragments, implying that amurin-9KY might act on the bacterial membrane and induce disruption of membrane integrity. Ampicillin-treated *S. aureus* exhibited no significant morphological alteration compared with the untreated bacteria (Figure 5C), except for the appearance of many granules on the surfaces. Ampicillin usually kills bacteria by disturbing the synthesis of the cell wall.

A: *S. aureus* ATCC25923 control; B: Amurin-9KY-treated *S. aureus* ATCC25923; C: Ampicillin-treated *S. aureus* ATCC25923.

## DISCUSSION

Among the AMP families identified from ranid species, the C-terminal heptapeptide ring (Rana box domain, two cysteine residues connected by a disulfide bridge) is a common structural feature, which broadly exists in families such as brevinin-1, brevinin-2, esculentin-1, esculentin-2, ranatuerin-1, ranalexin, japonicin-1, nigrocin-2, odorranain-C, odorranain-D, odorranain-F, odorranain-G, odorranain-H, odorranain-P1 and hainanenin (Conlon, 2004; Duda et al., 2002; He et al., 2012; Li et al., 2007; Matutte et al., 2000). In addition, other cyclic ranid AMPs have also been identified, including those with a C-terminal octapeptide ring (japonicin-2), C-terminal hexapeptide ring (ranatuerin-2, amolopin-6), middle heptapeptide ring (palustrin-2) or ring formed by more than seven amino acids (odorranain-A, odorranain-B, odorranain-J, odorranain-P2, odorranain-T, odorranain-U and ranacyclins) (Conlon, 2004; Duda et al., 2002; He et al., 2012; Li et al., 2007; Matutte et al., 2000). To date, three AMP families characterized with C-terminal amidation have been identified from ranid frogs, including temporins, ranacyclins and tiannanenin (Conlon, 2004; He et al., 2012; Li et al., 2007). Among the AMP families identified from ranid frogs so far, ranacyclin is a unique family possessing both a disulfide bridge-formed ring and amidated C-terminus (Mangoni et al., 2003). The ring structure of ranacyclins is composed of 11 residues and is located in the middle of the sequences. Comparatively, the novel AMP in the present study (amurin-9KY) is the first reported to have both a C-terminal heptapeptide ring and amidated C-terminus.

The three derivatives (amurin-9KY1–3) were designed here to evaluate the influence of C-terminal amidation and the C-terminal heptapeptide ring on the biological activities of amurin-9KY. The four peptides were chemically synthesized and their *in vitro* functions were subsequently examined. The antimicrobial assay results indicated that C-terminal amidation played an important role in the antimicrobial activity of amurin-9KY, whereas the heptapeptide ring contributed no obvious influence. SEM demonstrated that amurin-9KY induced obvious bacterial membrane morphological alteration, indicating that it might act through the disruption of bacterial membrane integrity.

Amurin-9KY possessed strong antimicrobial activity against gram-positive bacteria and low hemolytic activity, consistent with the features of temporin family AMPs. Previous study of temporin-1Od from the Japanese mountain brown frog *R. ornativentris* demonstrated that it possessed high antimicrobial potency towards *S. aureus* due to a positive charge associated with the free N-terminal amino group (Kim et al., 2001). The current study further confirmed that the free amino group is essential for the antimicrobial activity of AMPs, no matter at which terminus (N or C) it is located. Previous structure-activity analysis of brevinin 1E, a brevinin-1 family AMP identified from *R. esculenta*, demonstrated that the elimination of the intra-disulfide bridge did not greatly affect the antimicrobial activity (Kwon et al., 1998), which was further verified in the present result.

Regarding hemolytic activity, both C-terminal amidation and the heptapeptide ring significantly reduced the hemolytic activity of amurin-9KY compared with the derivatives without these two structural features. Elimination of the C-terminal heptapeptide ring greatly increased the anti-oxidant activity of amurin-9KY, whereas elimination of C-terminal amidation did not affect it at all. These results are likely because the unpaired thiol group of cysteine generated after elimination of the heptapeptide ring acted as an electron donator to the radical, which is crucial for the anti-oxidant activity of peptides (Akerström et al., 2007; Liu et al., 2010).
